# Survival and mortality predictors among children living with HIV initiating antiretroviral therapy in Angola: a retrospective cohort study

**DOI:** 10.1186/s12889-026-27747-w

**Published:** 2026-05-11

**Authors:** Paulo Ney Solari, Pedro Aguiar, Gonçalo Figueiredo Augusto

**Affiliations:** 1 NOVA National School of Public Health, NOVA University Lisbon, Av. Padre Cruz, Lisbon, 1600-407 Portugal; 2 Comprehensive Health Research Centre (CHRC), NOVA University Lisbon, Rua do Instituto Bacteriológico 5, Edifício Amarelo, Lisbon, 1150-082 Portugal; 3 Public Health Research Centre (CISP), NOVA National School of Public Health, NOVA University Lisbon, Lisbon, Portugal; 4 National Institute for Health Research of Angola (INIS), Rua Amílcar Cabral 96, Luanda, Angola

**Keywords:** Paediatric HIV, Mortality, Predictors, Angola, Antiretroviral therapy

## Abstract

**Background:**

Children living with HIV continue to face high mortality rates in resource-limited settings, especially in sub-Saharan Africa. In Angola, there is a lack of robust data to guide clinical and programmatic strategies targeting this vulnerable population. This study aimed to estimate survival and identify predictors of mortality in children with HIV followed up at a paediatric referral hospital in Angola.

**Methods:**

A retrospective cohort study of children (≤ 15 years) who started ART between 2016 and 2018, with follow-up until 2023. Kaplan–Meier curves and Cox regression models were used to estimate survival and identify factors associated with death.

**Results:**

During 1,577 person-years of follow-up, a total of 420 deaths occurred, corresponding to a mortality rate of 26.6 deaths per 100 person-years. The median follow-up time was 11.9 months (interquartile range: 3.4–38.5). Median survival was significantly lower among children with non-vertical transmission, WHO clinical stage III/IV, CD4 counts below the age-specific threshold, underweight status, and opportunistic infections. In the multivariable Cox model including transmission route, WHO clinical stage, baseline CD4 count, underweight status, and age group, non-vertical transmission (AHR 1.35, 95% CI 1.11–1.65), WHO clinical stage III/IV (AHR 1.82, 95% CI 1.36–2.46), and CD4 below the age-specific threshold (AHR 1.82, 95% CI 1.06–3.12) remained independent predictors of mortality.

**Conclusion:**

The high mortality observed in this cohort was associated with advanced clinical severity at admission. The finding that non-vertical transmission was independently associated with mortality suggests the need for improved screening and tailored care strategies for children and adolescents infected outside the mother-to-child context. Strengthening early diagnosis and timely initiation of ART is crucial to reducing preventable deaths and achieving global paediatric HIV control targets.

**Supplementary Information:**

The online version contains supplementary material available at 10.1186/s12889-026-27747-w.

## Background

HIV/AIDS is still one of the leading causes of child morbidity and mortality worldwide, especially in low- and middle-income countries such as Angola. Globally, in 2023 alone, 1.4 million children aged 0 to 14 had HIV (3% of those infected) [1].

Antiretroviral treatment (ART) has advanced significantly in recent years, but data show that only 57% of children living with HIV received treatment, compared to 77% of adults. This certainly draws our attention to paediatric care [1]. It should not be forgotten that this disparity can be seen in clinical results, i.e. in terms of mortality. In 2023, children accounted for 12% of all deaths related to HIV/AIDS, even though they represented only 3% of those infected, as described above [2].

Despite these findings, it should be noted that globally, since 2010, new HIV infections have been declining among children, more specifically from 300,000 to 120,000 annually. Even so, paediatric HIV remains a major public health concern, particularly in resource-limited settings [1].

Sub-Saharan Africa has the highest paediatric HIV burden, accounting for the majority of infections and deaths among children. To reduce this impact, action must be taken across the entire care continuum: early diagnosis, immediate initiation of ART and paediatric-specific retention strategies [2].

In Ethiopia, a multicentre study estimated mortality at 2.53 per 100 child-years, with higher risk in the presence of opportunistic infections, malnutrition and advanced clinical stage according to WHO classification [3]. Another study conducted in Cameroon showed a lower probability of survival in children who were in WHO clinical stages II and IV at the start of ART [4].

Overall, cohort studies conducted in Nigeria, Cameroon, and Zambia showed that the survival of children on ART was associated with good treatment adherence, nutritional support, and the absence of comorbidities [4–6]. Studies from southern Africa have also drawn attention to the underreporting of paediatric deaths from HIV, with up to one-third of deaths not recorded by formal health systems [7].

The results of the EARTH Study [8], conducted in several African countries, further reinforce this concern, revealing that even infants who start ART early continue to experience very high mortality rates, emphasising that early ART, while essential, is insufficient in contexts of extreme social and clinical vulnerability. These results highlight persistent structural and clinical challenges to paediatric survival despite increased ART coverage and call for context-specific systematic investigations.

What we mean is that despite the growing number of studies on paediatric HIV in African settings, there are still critical gaps in knowledge, particularly in relation to countries such as Angola, where published cohort evidence remains limited. Most existing studies have been conducted in settings such as Ethiopia, South Africa, Nigeria and Zambia, where access to laboratory services, early diagnosis and ART coverage may be comparatively higher than in under-represented settings such as Angola [3–6].

In addition to global and regional trends, the Angolan context presents specific programmatic and epidemiological characteristics that directly relate to known predictors of mortality. Available national reports and programme data highlight ongoing challenges, including gaps in early infant diagnosis, late presentation, and limited access to paediatric laboratory monitoring [9–11], all of which are factors associated with advanced WHO stage, severe immunosuppression, and poorer survival outcomes [3, 12, 13]. These system-level constraints are particularly evident at the national referral hospital where this cohort was recruited, which receives severe and complex cases from several provinces.

It should also be noted that many studies [3, 12, 13] suffer from methodological limitations, including small sample sizes, short follow-up periods, inadequate control of confounding factors and reliance on hospital data. These limitations restrict the generalisation of results to broader paediatric populations living in contexts with weaker health systems and different sociocultural and epidemiological characteristics.

In Angola, HIV testing and prevention of vertical transmission of HIV are organised within a national programme led by the National Institute for the Fight against AIDS (INLS). The 2023 National HIV Testing and Counselling Protocol recommends provider-initiated HIV testing as a routine component of antenatal care, childbirth, postnatal and paediatric services, and includes specific algorithms for early infant diagnosis and retesting of HIV-negative pregnant and breastfeeding women [9].

The most recent national ART guidelines have adopted a nationwide “test and treat” strategy since 2019, whereby all individuals diagnosed with HIV initiate ART irrespective of CD4 cell count, and since 2021 Angola has transitioned to dolutegravir-based first-line regimens [10].

This approach is consistent with WHO recommendations that pregnant and breastfeeding women living with HIV should start lifelong triple-drug ART (Option B+) and that HIV testing during pregnancy and breastfeeding should be repeated at several time points to detect incident infection and reduce vertical transmission [14].

Previous analyses of vertical transmission services in Angola have documented a rapid expansion in the number of vertical transmission sites and HIV tests performed in antenatal care, but also persistent gaps in coverage of ART and early infant diagnosis for HIV-exposed infants, underscoring the need to better understand paediatric outcomes in this setting [11].

In Angola, no cohort studies have yet been published that robustly assess predictors of mortality or long-term survival among children living with HIV. Available data are often aggregated, outdated, and rarely disaggregated by clinical, immunological, or contextual risk factors. This lack of local evidence hinders the development of data-driven public health policies, national clinical protocols, and the prioritisation of interventions for high-risk subgroups, such as children with malnutrition, opportunistic infections, or critically low CD4 counts.

We believe there is an urgent need for research that employs a robust methodology to document survival patterns and factors associated with mortality in this vulnerable population. In doing so, the present study aims to fill a significant scientific gap, inform clinical decision-making, improve HIV programme management, and support Angola’s commitment to the UNAIDS 2030 goal of ending AIDS as a public health threat.

This study aimed to estimate survival and identify predictors of mortality among children living with HIV who were followed up at a paediatric referral hospital in Angola. Children enrolled between 2016 and 2018 were included, with follow-up until 2023, allowing for a minimum of five years of potential observation.

## Materials and methods

### Study design

This is a retrospective observational cohort study. The cohort is considered *closed* because all eligible children were enrolled during a fixed inclusion period (January 2016 to December 2018), with no additional participants entering after this baseline window. Once enrolled, all children were followed until death, loss to follow-up, transfer to another facility, or administrative censoring in 2023.

### Location and period of study

The study was conducted at the David Bernardino Paediatric Hospital (HPDB) in Luanda, the capital of Angola, which serves as the main national referral centre for the diagnosis, treatment and follow-up of children living with HIV. The hospital has a multidisciplinary team, a specialised outpatient clinic and a laboratory to support the clinical follow-up of paediatric HIV cases.

This hospital receives cases from several provinces, including newly diagnosed cases, cases restarting ART and cases of non-vertical transmission.

Data were obtained from routine clinical and laboratory records of children who started antiretroviral treatment between 1 January 2016 and 31 December 2018, with documented follow-up until 31 December 2023. As a national referral centre cohort, the severity at entry is higher than in primary-care or district-level cohorts, which may limit the external generalizability of the findings.

### Study population

The study population consisted of children aged 15 years or younger with a confirmed diagnosis of HIV infection who started ART at HPDB during the inclusion period and were followed up until 31 December 2023.

Children with a confirmed diagnosis of HIV (positive PCR test for those under 18 months or two reactive rapid tests for those aged 18 months or older) who started ART between 1 January 2016 and 31 December 2018 and who had at least one follow-up clinical record after treatment initiation were included.

Children with no information on the date of ART initiation, those transferred to another facility before any follow-up visit, or those without information on vital status at the end of follow-up were excluded.

Children with less than one month of follow-up remained in the analysis to ensure full capture of time at risk since ART initiation.

The age range up to 15 years allowed the inclusion of both children with congenital infection and adolescents potentially infected through behavioural transmission or sexual violence.

The route of HIV transmission was classified based on information documented in the clinical records at the time of diagnosis. Vertical transmission was defined as HIV infection acquired from the mother during pregnancy, delivery, or breastfeeding, as documented in the medical file according to national diagnostic criteria. Non-vertical transmission was assigned only when the clinical record explicitly documented alternative exposure pathways, such as sexual abuse, unsafe medical procedures, blood transfusion, or other non-maternal exposures in older children.

### Data source and collection procedures

The data used in this study were obtained from an electronic database of the HPDB that integrates clinical, laboratory, anthropometric, and therapeutic information on all children followed up at the unit.

Data collection was performed retrospectively, based on clinical and laboratory records systematically archived between 1 January 2016 and 31 December 2023. Sociodemographic variables, clinical data at the start of ART, laboratory results, nutritional status assessment and clinical evolution throughout follow-up were extracted.

### Study variables and operational definitions

Sociodemographic, clinical, laboratory, nutritional, and therapeutic variables were collected. The dependent variable considered was the time to death after initiation of ART. The independent variables and their operational definitions are described below.

### Outcome and censoring

The main outcome of the study was time to death, measured in months, defined as the interval between the date of initiation of ART and the date of clinical death documented in the records.

Children with no record of death by 31 December 2023 were considered censored. Children transferred to another unit or lost to follow-up were also classified as censored, and the follow-up time was calculated as the number of months between the date of initiation of ART and the date of death or last recorded visit.

Mortality incidence rate was calculated by dividing the number of deaths by the total person-time at risk.

### Independent variables

#### Sociodemographic

These included age at the start of ART (recorded in full years and later grouped according to sample distribution: ≤5 years and > 5 years), the sex of the child (male or female) and the primary caregiver, understood as the person who accompanies the child to appointments and ensures clinical follow-up, categorised as biological parent or other caregiver (e.g. grandmother, uncle or institutional guardian).

#### Clinical

The clinical stage of HIV infection was analysed according to the WHO classification (grouped into I/II or III/IV, according to the signs and symptoms recorded in the clinical record), and the presence of opportunistic infections, defined as the record of at least one diagnosed condition, such as tuberculosis, oral or oesophageal candidiasis, pneumocystosis or recurrent herpes.

#### Laboratory

CD4 counts were measured at the hospital laboratory using routine flow cytometry assays, according to standard national procedures in place during the study period. CD4 values were analysed according to the thresholds defined for severe immunosuppression: values below 25% in children under 5 years of age or below 350 cells/mm³ in children aged 5 years or older, and subsequently categorised as “below” or “above” these thresholds. Baseline haemoglobin was classified into two groups: <10.0 g/dL, indicating moderate or severe anaemia, and ≥ 10.0 g/dL, considered normal or slightly decreased.

#### Nutritional status

was assessed according to WHO Child Growth Standards and WHO Growth Reference for school-aged children and adolescents. Weight and height were measured during routine clinical visits using standard calibrated scales and stadiometers available at the hospital. For children younger than 10 years, malnutrition was defined using the weight-for-age Z score (WAZ), with WAZ < − 2 SD classified as underweight. For children aged 10 to 15 years, BMI-for-age Z score (BAZ) was used, with BAZ < − 2 SD indicating thinness [15, 16]. These indicators were extracted directly from the clinical record as routinely assessed at HPDB.

### Statistical analysis

All statistical analyses were performed using IBM SPSS Statistics, version 29.0.2 (IBM Corp., Armonk, NY, USA). Survival time was defined as the number of months between the start of ART and the occurrence of the event or censoring. The event of interest was death from any cause. Censoring occurred when a child was transferred to another facility, lost to follow-up, or alive at the administrative end of the study period (31 December 2023). The significance level (α) was set at 0.05 for all analyses, and all tests were two-tailed.

No time-dependent covariates were included in the main Cox regression model. The proportional hazards assumption was assessed through graphical inspection of log–log survival plots and by testing time-dependent covariates created by interacting each predictor with the logarithm of follow-up time.

In the descriptive analysis, continuous variables were summarised by mean and standard deviation (SD) when they had an approximately normal distribution, while categorical variables were presented in absolute and relative frequencies (%).

Survival probabilities at 1, 2, 3, 4 and 5 years were estimated using the Kaplan–Meier method. All children were included in the denominator for each estimate, regardless of follow-up duration, with censoring applied to those who did not complete the time interval. Therefore, the 5-year survival estimates are based on the full cohort and not only on children with ≥ 5 years of follow-up.

Although the median follow-up time was 11.9 months, the Kaplan–Meier method allows estimation of survival probabilities beyond the median follow-up because individuals contribute person-time until the occurrence of the event or censoring. As follow-up progresses, the number of children at risk decreases due to censoring and deaths, and survival estimates at later time points are therefore based on progressively smaller risk sets.

Comparisons between groups were made using the log-rank test. Cox regression (proportional hazards model) was used to estimate crude hazard ratios (HR) and adjusted hazard ratios (aHR), each with their respective 95% confidence intervals (95% CI). Variables were considered for inclusion in the multivariable Cox regression model if they showed a p-value < 0.25 in the bivariate analysis or were considered clinically relevant based on previous literature. All candidate variables were then entered into the multivariable model. Collinearity among predictors was assessed using the variance inflation factor (VIF) to ensure that no significant multicollinearity was present.

The overall adequacy of the final model was also evaluated by examining the stability and plausibility of hazard ratio estimates, as well as the consistency between crude and adjusted associations.

No variable transformation was necessary, and no outliers were removed. A complete case analysis approach was used. Records were excluded when essential information for survival analysis was missing, namely, date of ART initiation, follow-up visits, or documented vital status, because the absence of these data made it impossible to calculate survival time or determine the event. The missing data were therefore structural rather than sporadic, and no imputation was performed. Because the analytic cohort was defined after these exclusions, all variables included in the regression models had complete data for the final sample.

Baseline CD4 measurements were obtained from routine laboratory records available in the hospital database. Because the analysis was restricted to complete cases with documented baseline clinical and laboratory information, the classification of severe immunosuppression was based on the available CD4 values recorded at ART initiation.

The sample size was defined by enumerating all eligible children who met the inclusion criteria between 2016 and 2018, without prior calculation of the sample size.

### Ethical considerations

The study was conducted in accordance with the ethical principles set out in the Declaration of Helsinki (revised in 2024) and in accordance with national regulations governing health research in Angola. The protocol was reviewed and approved by the Ethics Committee of the Angolan Ministry of Health (reference No. 10/CEMS/2024) and obtained authorisation from the Scientific Directorate of the HPD.

Since the study was based exclusively on previously recorded and duly anonymised secondary data, the requirement for individual informed consent was formally waived. Nevertheless, all data were treated with the utmost rigour with regard to confidentiality, anonymity and the safeguarding of sensitive information about participants.

Access to the database was restricted to the research team, subject to institutional authorisation, and all results were presented in aggregate form, without any possibility of individual identification.

### Use of AI tools

Certain sections of the manuscript were edited with the assistance of a large language model (ChatGPT, OpenAI), used solely to improve language clarity and style. All scientific content, data interpretation, and conclusions were fully developed and verified by the authors.

## Results

**Fig. 1:**
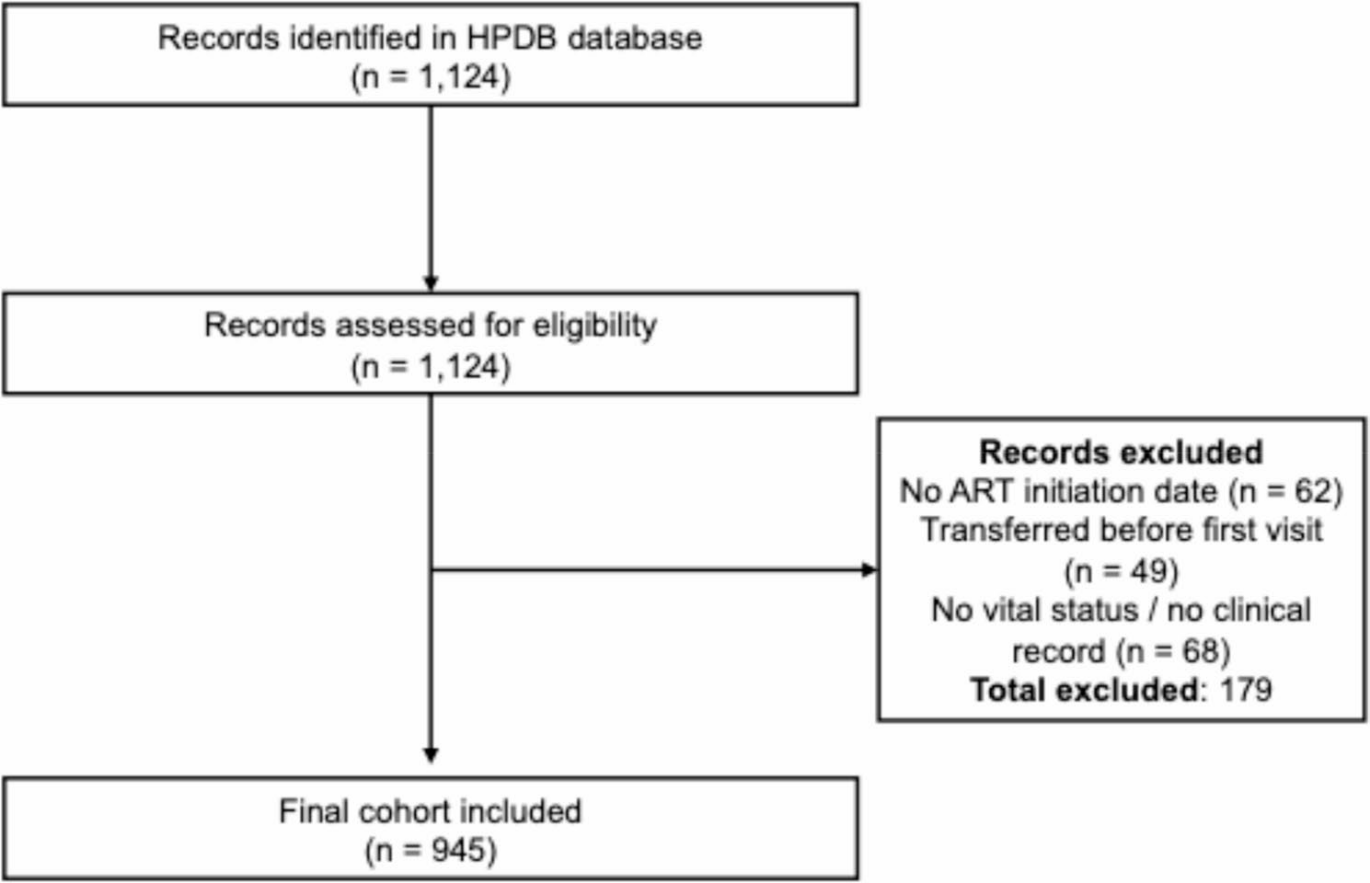
Flow diagram of participant selection for the retrospective cohort

A total of 1,124 records of children with confirmed HIV infection were identified in the HPDB database for the period 2016–2018. After verifying eligibility conditions, 179 records were excluded: 62 due to absence of ART initiation date, 49 corresponding to children transferred to another facility before any follow-up visit, and 68 lacking vital status information or any clinical record after treatment initiation. The final cohort therefore comprised 945 children, all with documented ART initiation and clinical follow-up until 31 December 2023 (Fig. 1).

At the start of ART, 570 children (60.3%) were five years old or younger, and 477 (50.5%) were male. In 771 cases (81.6%), the primary caregiver was a biological parent, and the route of transmission was predominantly vertical, observed in 598 children (63.3%).

Regarding the initial clinical status, 650 (68.8%) were in WHO clinical stage I or II, while 27 (2.9%) showed severe immunosuppression according to age-based CD4 count criteria. Haemoglobin was below 10 g/dL in 180 children (19%) and 114 (12.1%) had moderate to severe malnutrition (weight-for-age Z-score < -2 SD). Opportunistic infections were documented in 341 children (36.1%) at the time of inclusion. During follow-up, there were 420 deaths, corresponding to a cumulative mortality proportion of 44.4% (Table 1).

**Table 1:** Sociodemographic and clinical characteristics

	*n*	%
Age (years)		
Mean (SD)	5.8 (4.1)	
<= 5	570	60.3
> 5	375	39.7
Sex		
Male	477	50.5
Female	468	49.5
Primary caregiver		
Parents	771	81.6
Relatives	174	18.4
Transmission		
Vertical	598	63.3
No-vertical	347	36.7
WHO clinical stage		
Stage I and II	650	68.8
Stage III and IV	295	31.2
CD4 Count		
Below threshold	27	2.9
Above threshold	918	97.1
Haemoglobin		
≥ 10 g/dL	765	81.0
< 10 g/dL	180	19.0
Underweight		
No	831	87.9
Yes	114	12.1
Opportunistic disease		
No	604	63.9
Yes	341	36.1
Outcome		
Censored	525	55.6
Death	420	44.4

*n* represents the number of children in each category; % was calculated using the total cohort size (*N* = 945) as the denominator

Early mortality was substantial. Kaplan–Meier estimates showed that cumulative mortality reached 28.4% at 3 months, 31.3% at 6 months, and 36.7% at 12 months after ART initiation, indicating that a large proportion of deaths occurred during the first year of follow-up.

The mortality incidence rate was 26.6 per 100 person-years, calculated by dividing the total number of deaths (420) by the total person-years of follow-up (1577). The median follow-up time was 11.9 months (ranging from 0 to 59.6 months).

In the survival analysis (Table 2), children aged ≤ 5 years had a longer median survival (52.2 months; 95% CI: 46.1–58.3) and a higher proportion of survival at 5 years (33.0%) than those aged over 5 years (42.5 months; 19.8%; *p* = 0.031).

Gender did not show a significant association with survival time (*p* = 0.662). Children accompanied by their parents had shorter survival times (43.3 months) compared to those accompanied by other caregivers (55.9 months; *p* < 0.001).

Vertical transmission was associated with longer survival (37.3 vs. 28.1 months; *p* < 0.001), as was WHO clinical stage I/II (50.0 vs. 30.9 months; *p* = 0.013).

Lower median survival rates were observed in children with CD4 below the age threshold (9.0 months; *p* < 0.001), with haemoglobin < 10 g/dL (30.9 months; *p* = 0.133), with malnutrition (7.5 months; *p* < 0.001) and opportunistic infection (10.0 months; *p* < 0.001).

Survival time and proportions according to baseline characteristics are presented in Table 2.

**Table 2:** Survival time and proportions

Variable	Categories	Survival at 5 years (%)	Median survival (months, 95% CI)	Log-rank *p*-value
Age (years)	≤ 5	33.0	52.2 (46.7–57.6)	0.031
	> 5	19.8	42.5 (33.0–52.1)	
Sex	Male	30.3	49.2 (40.3–58.1)	0.662
	Female	19.3	48.7 (40.4–57.0)	
Primary caregiver	Parents	12.9	43.3 (35.3–51.2)	< 0.001
	Relatives	15.6	55.9 (47.3–64.5)	
Transmission	Vertical	29.6	37.3 (35.2–39.5)	< 0.001
	Non-vertical	23.8	28.1 (24.9–31.2)	
WHO clinical stage	I & II	30.1	50.0 (43.6–56.4)	0.013
	III & IV	19.9	30.9 (4.1–57.6)	
CD4 count	Below threshold	7.5	9.0 (6.5–11.5)	< 0.001
	Above the threshold	13.0	49.2 (43.0–55.3)	
Haemoglobin (g/dL)	≥ 10 g/dL	52.1	50.0 (43.6–56.4)	0.133
	< 10 g/dL	30.1	30.9 (4.1–57.6)	
Underweight	No	15.5	54.8 (51.7–57.9)	< 0.001
	Yes	2.3	7.5 (5.0–10.5)	
Opportunistic disease	No	15.2	56.0 (53.8–58.1)	< 0.001
	Yes	14.5	10.0 (6.0–13.5)	

Kaplan–Meier survival probabilities by baseline characteristics. Denominators include all 945 children; censoring was applied according to individual follow-up time

**Fig. 2:**
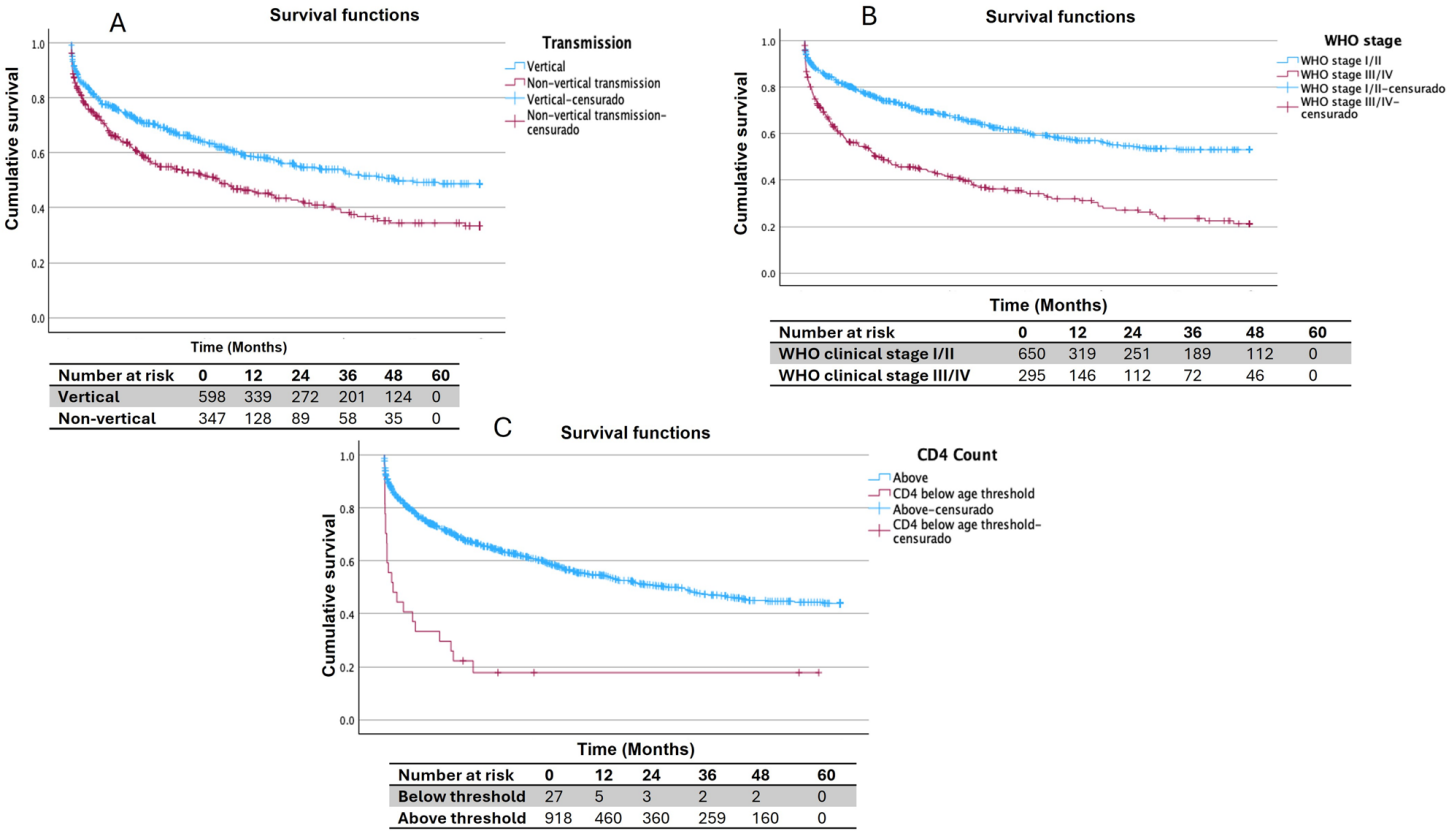
Kaplan–Meier survival curves of children living with HIV after ART initiation, stratified by key baseline characteristics. **A** Survival according to route of HIV transmission (vertical vs. non-vertical). **B** Survival according to WHO clinical stage at ART initiation (stage I/II vs. stage III/IV). **C** Survival according to baseline CD4 count, classified as below or above the age-specific threshold. Time is expressed in months since ART initiation. The y-axis represents cumulative survival probability. Tick marks indicate censored observations. Across all panels, lower survival probabilities were observed among children with non-vertical transmission, WHO clinical stage III/IV, and CD4 count below the age-specific threshold. Differences between groups were assessed using the log-rank test

Kaplan–Meier survival curves according to transmission route, WHO clinical stage, and baseline CD4 count are shown in Fig. 2. Children with non-vertical transmission, WHO clinical stage III/IV, and CD4 counts below the age-specific threshold had lower probabilities of survival over time. The subgroup of children with CD4 counts below the age-specific threshold was small, reflecting the low proportion of severe immunosuppression at the start of ART.

Table 3 presents both crude and adjusted hazard ratios for the predictors included in the Cox regression analysis. In the multivariable Cox regression analysis, non-vertical transmission remained significantly associated with increased mortality risk (aHR: 1.35; 95% CI: 1.11–1.65; *p* = 0.003) compared with vertical transmission. Children classified as WHO clinical stage III/IV also showed a substantially higher risk of death (aHR: 1.82; 95% CI: 1.36–2.46; *p* = 0.001) compared with those in Stages I/II. Severe immunosuppression, defined as a CD4 count below the age-specific threshold, remained an independent predictor of mortality (aHR: 1.82; 95% CI: 1.06–3.12; *p* = 0.029).

**Table 3:** Independent predictors of mortality in HIV-infected children

	CHR (95% CI)	aHR (95% CI)	*p*
Transmission			
Non-vertical	1.626 (1.337–1.975)	1.35 (1.11–1.65)	0.003
Vertical	1	1	
WHO clinical stage			
Stage III/IV	1.19 (0.94–1.51)	1.82 (1.36–2.46)	0.001
Stage I/II	1	1	
CD4 Count			
Below threshold	2.25 (1.38–3.36)	1.82 (1.06–3.12)	0.029
Above threshold	1	1	
Underweight			
Yes	5.62 (4.42–7.08)	1.31 (0.71–1.86)	0.230
No	1	1	
Age (> 5 years)			
> 5 years	1.20 (1.00–1.41)	1.11 (0.90–1.36)	0.310
≤ 5 years	1	1	

*cHR* Crude Hazard Ratio, *aHR* Adjusted Hazard Ratio, *95% CI* 95% Confidence Interval

The proportional hazards assumption was formally tested and no statistically significant violations were identified.

## Discussion

In this national referral cohort, mortality was high, consistent with severe clinical presentation at entry and structural barriers to timely diagnosis and treatment. Advanced WHO stage, severe immunosuppression, and non-vertical transmission were independently associated with a higher risk of death. These are associations rather than causal effects.

The mortality observed in this study is considerably higher than that reported in other African cohorts receiving antiretroviral treatment. Early mortality was particularly pronounced, with a substantial proportion of deaths occurring within the first months after ART initiation. This figure should therefore be interpreted in light of the specific context of the population studied. Although routine national programme reports generally describe lower mortality estimates, these figures often derive from surveillance systems with incomplete death reporting and a lower concentration of advanced cases than those managed at a national referral centre such as HPDB.

The elevated mortality observed in this cohort is also consistent with the referral patterns at HPDB. As the country’s sole national paediatric referral centre with dedicated HIV inpatient services, HPDB receives a concentration of clinically severe and late-presenting cases from multiple provinces. This is reflected in our baseline profile, which shows high frequencies of WHO stage III/IV disease, severe immunosuppression, opportunistic infections, and malnutrition at ART initiation. These indicators support referral bias as a key driver of the high mortality observed. Although national programme reports describe lower mortality, these estimates derive from routine surveillance systems with incomplete death reporting and far fewer advanced cases than those managed at HPDB.

This is a hospital cohort composed of children up to 15 years of age, followed up at a national paediatric referral hospital located in the country’s capital, which systematically receives the most severe cases from different provinces. This centralisation of care, combined with the fact that many of these children or their caregivers only seek help at advanced stages of infection, results in a more severe initial clinical profile, with a high frequency of immunosuppression, opportunistic infections and malnutrition, all of which are recognised as risk factors for mortality, especially in resource-limited settings such as Angola.

A multicentre study by Nyakato et al. [7], for example, which tracked 680 children and young people with HIV (< 25 years) in five countries in southern Africa, revealed that 9.1% were dead, with 44% of these deaths occurring in the first three months after their last consultation.

Contrary to this reality, our cohort is based on complete clinical records, with follow-up until the outcome occurred, which ensures greater accuracy in mortality estimates. Thus, the high mortality observed may reflect not only the limitations of the health system but also the methodological quality of the study in capturing deaths.

Our findings are consistent with Tagarro et al. [8], who followed infants starting ART before 3 months of age in South Africa, Mozambique, and Mali, observing cumulative mortality of 10% at 12 months and 12% at 24 and 36 months; most deaths occurred in the first six months.

Similar results were reported by Moshi et al. [17] in a paediatric cohort study in southern Tanzania (KIULARCO, 2005–2023), where 1,089 children were followed for a median of 3.3 years, with 130 deaths (12%). The cumulative incidence of mortality was 8% at 1 year (95% CI: 6–10) and 14% at 5 years (95% CI: 12–17). Considering the combined outcome of death/loss to follow-up, the probabilities reached 26% at 1 year and 46% at 5 years. In Cox models for the combined outcome, the risk was higher with severe HIV disease at inclusion (aHR 1.54; 95% CI 1.26–1.89), age < 5 years (aHR 1.28; 95% CI 1.01–1.66), hospitalisation (aHR 1.93; 95% CI 1.42–2.63), residence ≥ 50 km (aHR 1.72; 95% CI 1.37–2.16), and delayed ART initiation (aHR 3.40; 95% CI 2.70–4.27), while enrolment in more recent years was protective (aHR 0.51; 95% CI 0.37–0.70 for 2017–2023 vs. 2005–2012). For isolated mortality, the predictors were broadly similar, except for age and distance, which did not maintain an association.

Two other key predictors in this study were advanced clinical stage (WHO III/IV) and severe immunosuppression, measured by CD4 below the age threshold. The relatively low proportion of severe immunosuppression observed at baseline should be interpreted cautiously. This may partly reflect the use of strict WHO age-specific thresholds as well as the reliance on routine clinical records in a retrospective cohort.

Both showed a strong association with the risk of death. These findings suggest that many children may have been diagnosed and initiated on treatment at advanced stages of disease.

These results coincide with those described by Mashiri et al. [18] in South Africa, who showed that lower baseline CD4 counts (median of 60 cells/mm³ at death) were strongly associated with increased mortality. In their multivariable model, CD4 was analysed as a continuous variable, with each unit increase associated with a small but significant reduction in mortality risk (aHR per cell increase = 0.99; *p* = 0.02). Advanced clinical stage was the factor most strongly associated with early mortality in that study. Similarly, Tessema et al. [19] in Ethiopia found that clinical stage III/IV doubled the risk of death (AHR = 2.6), and low CD4 levels were also significantly associated with the outcome (*p* = 0.001).

Gurmu et al. [20] analysed 286 children in a retrospective cohort and found that CD4 below the threshold (AHR 3.7; 95% CI: 1.4–9.5; *p* = 0.006) and WHO clinical stage III (AHR 3.3; 95% CI: 1.2–7.4; *p* = 0.002) and IV (AHR 5.4; 95% CI: 1.5–19.8; *p* = 0.010) were independently associated with mortality.

In Kenya, Nyandiko et al. [21] reinforced these findings, reporting an increased risk of mortality among children with WHO clinical stage III (aHR 2.63; 95% CI 1.12–6.18), while stage IV showed only a non-significant trend (aHR 2.20; 95% CI 0.94–5.18). Higher CD4 counts (> 350 cells/mm³) were associated with a protective effect in the multiple imputation analysis (aHR 0.64; 95% CI 0.44–0.93), although this was not confirmed in the complete case model. Older age (≥ 11 years) was associated with a substantially higher risk of mortality (aHR 8.36; 95% CI 3.60–19.40).

In our study, the same patterns were observed. Children who started ART at an advanced clinical stage and with severe immunosuppression had a high risk of death, especially in the first months of follow-up. This result reinforces the urgent need for early testing, rapid linkage to care, and timely initiation of ART, especially in regions where late diagnosis remains a serious obstacle.

Another relevant aspect was the role of non-vertical transmission as an independent predictor of mortality, a finding that has been little explored in the literature but which our study was able to demonstrate quantitatively. In this cohort, the proportion of non-vertical transmission was unexpectedly high. Although the specific circumstances of infection could not be directly documented, this pattern likely reflects delayed HIV diagnosis outside the prevention-of-vertical-transmission context, with children being identified only after presenting with advanced disease.

Such delays may be associated with vulnerabilities related to child protection, gaps in infection control practices, or insufficient coverage of HIV testing beyond the perinatal period. Irrespective of the exact mechanisms, the magnitude of non-vertical transmission observed in this cohort may reflect systemic weaknesses in early case detection and prevention, and underscores the need to strengthen child safeguarding frameworks, improve the safety of healthcare and transfusion services, and expand routine HIV testing strategies targeting older children and adolescents.

Importantly, age was included as a covariate in the multivariable Cox model and did not remain independently associated with mortality. This indicates that the observed association between non-vertical transmission and mortality is not merely a proxy for older age, but reflects additional vulnerability factors such as delayed diagnosis, complex exposure pathways, and late engagement with HIV care.

Although few studies explicitly model the route of HIV acquisition, available multicentre evidence suggests that infections acquired outside the perinatal period are associated with worse outcomes. The IeDEA global cohort analysis (61,242 patients; 34 countries) did not directly classify transmission route; instead, it used age at entry into care (≥ 15 years) as a proxy for infection acquired during adolescence. This approach is not fully comparable to the present study, nonetheless, the IeDEA findings—higher mortality (5.4% vs. 3.9%) and markedly higher loss to follow-up (69% vs. 26%) among those entering care at ≥ 15 years—suggest that individuals infected outside early childhood may experience more unstable treatment trajectories and poorer outcomes, consistent with broader evidence from adolescent HIV cohorts. Similarly, a South African cohort reported that adolescents with sexually acquired infection had less consistent adherence patterns and lower rates of sustained viral suppression than those with perinatally acquired HIV, a plausible mechanism underlying the higher mortality observed in non-vertical infections [22, 23].

Our data therefore fills an important gap by highlighting the need for differentiated screening and care strategies for children and adolescents infected outside the perinatal period, who are often excluded from traditional mother-child-centred approaches.

It is also worth noting that although malnutrition and opportunistic infections were associated with mortality in the univariate analysis, these associations did not persist after multivariable adjustment. This suggests that their effects may be largely explained by more advanced HIV disease severity, as captured by clinical stage and immunological status at baseline. In addition, limitations inherent to retrospective clinical records, such as incomplete documentation of anthropometric data or underreporting of opportunistic infections, may have contributed to the loss of statistical significance observed after adjustment. Nevertheless, both malnutrition and opportunistic infections remain clinically relevant conditions, and their absence from the final model should be interpreted with caution and not as a lack of importance in healthcare practice.

This study aimed to estimate survival and identify predictors of mortality in children living with HIV in Angola. The results showed that advanced clinical stage (III/IV), severe immunosuppression, and non-vertical transmission were independently associated with a higher risk of death, even after controlling for potential confounding factors.

These findings are consistent with evidence from other sub-Saharan African settings, which also point to late diagnosis and poor immune status as important determinants of mortality. The identification of non-vertical transmission as a predictor in this context adds new evidence to a limited body of literature and suggests the need for screening strategies and differentiated care for infected children and adolescents outside the perinatal period.

Future research should explore the mechanisms linking non-vertical transmission to worse outcomes, evaluate the effectiveness of differentiated care models, and test context-specific interventions that improve early diagnosis and timely treatment initiation.

### Limitations

This study has several limitations. Firstly, because it was conducted in a national referral hospital, the cohort includes a disproportionate number of severe and complicated cases, introducing referral bias that limits the generalisability of the findings to primary-care settings. Secondly, although essential variables such as ART initiation date, follow-up visits, and vital status were required for inclusion, other clinical and laboratory indicators were incompletely recorded in some files, which may have reduced the precision of certain estimates.

Thirdly, viral load data were not routinely available for a substantial proportion of the cohort and could therefore not be included in the analysis. Given that viral load is a well-established predictor of mortality among people living with HIV, its absence may have limited our ability to fully characterise disease severity and treatment response, potentially resulting in residual confounding.

Fourthly, despite adjusting for key clinical predictors, residual confounding cannot be excluded, particularly for factors not routinely documented in clinical records, such as adherence quality, socioeconomic vulnerability, breastfeeding exposure among older infants, and the timing of symptom onset. These unmeasured confounders may have influenced the observed associations.

Loss to follow-up and transfers were treated as non-informative censoring in the survival analysis, which is the standard assumption in Kaplan–Meier and Cox proportional hazards models. However, if some children lost to follow-up had died outside the hospital without documentation, mortality may have been underestimated.

Finally, the classification of non-vertical transmission relied on clinical documentation and may be prone to misclassification, particularly in cases with incomplete exposure histories or delayed diagnosis of vertically acquired infection.

In addition, baseline CD4 data were derived from routine laboratory documentation and may be subject to variability in clinical recording practices.

## Conclusion

In this retrospective cohort study with prolonged follow-up, conducted in a referral paediatric hospital in Angola, a high mortality rate was identified among children living with HIV, reflecting both clinical severity at admission and structural weaknesses in the health system.

Advanced clinical stage (WHO III/IV), severe immunosuppression, and non-vertical transmission were independently associated with mortality, even after controlling for potential confounders.

The identification of non-vertical transmission as an independent risk factor represents an innovative contribution with direct implications for the design of screening and retention strategies in care for paediatric populations that are often invisible in HIV policies.

These findings reinforce the urgent need for integrated interventions that combine early diagnosis, timely initiation of ART, and differentiated care according to transmission route and clinical-immunological profile. In resource-limited settings such as Angola, strengthening the response to paediatric HIV should be considered a strategic priority for reducing preventable mortality and meeting global targets for eliminating AIDS as a public health threat.

## Supplementary Information

Supplementary Material 1.

## Data Availability

The datasets analysed during this study are not publicly available due to confidentiality restrictions and the protection of participants’ clinical data. However, they may be made available by the corresponding author upon reasonable request and after authorisation from the David Bernardino Paediatric Hospital and the National Ethics Committee of Angola.

## Abbreviations

AHR: Adjusted Hazard Ratio
ART: Antiretroviral Therapy
CHR: Crude Hazard Ratio
CI: Confidence Interval
HIV: Human Immunodeficiency Virus
HPDB: Hospital Pediátrico David Bernardino
SPSS: Statistical Package for the Social Sciences
UNAIDS: Joint United Nations Programme on HIV/AIDS
WHO: World Health Organization
