# Supplementary material for: Survival and mortality predictors among children living with HIV initiating antiretroviral therapy in Angola: a retrospective cohort study
Source: BMC Public Health. 2026 May 11;26:2158. doi: 10.1186/s12889-026-27747-w (PMC13374198; doi:10.1186/s12889-026-27747-w)
Supplement: Supplementary file 1 — Supplementary Material 1. [file 12889_2026_27747_MOESM1_ESM.docx]

Supplementary **Table S1.** Kaplan–Meier Survival Probabilities at 1, 2, 3, 4 and 5 Years According to Baseline Characteristics

| Variable | Survival proportions | | | | |
| --- | --- | --- | --- | --- | --- |
|  | **1 Years (%)** | **2 Years (%)** | **3 Years (%)** | **4 Years (%)** | **5 Years (%)** |
| Age (years) |  |  |  |  |  |
| <= 5 | 59,6 | 56,7 | 54,4 | 47,9 | 33,0 |
| > 5 | 69,1 | 64,4 | 60,9 | 55,6 | 19,8 |
| Sex |  |  |  |  |  |
| Male | 63,2 | 60,3 | 57,6 | 50,8 | 30,3 |
| Female | 63,3 | 59,1 | 56,2 | 51 | 19,3 |
| Primary caregiver |  |  |  |  |  |
| Parents | 60,2 | 57,1 | 54,6 | 48,5 | 12,9 |
| Relatives | 76,8 | 71,4 | 67,3 | 61,8 | 15,6 |
| Transmission |  |  |  |  |  |
| Vertical | 69,6 | 65,8 | 62,9 | 56,2 | 29,6 |
| No-vertical | 52,0 | 48,7 | 46,1 | 41,2 | 23,8 |
| WHO clinical stage |  |  |  |  |  |
| Stage I e II | 65,3 | 61,4 | 58,8 | 52,1 | 30,1 |
| Stage III e IV | 54,8 | 52,6 | 48,8 | 45,4 | 19.9 |
| CD4 Count |  |  |  |  |  |
| Below threshold | 32,2 | 32,2 | 21,5 | 21,5 | 7,5 |
| Above threshold | 64,1 | 60,4 | 57,7 | 51,6 | 13,0 |
| Haemoglobin |  |  |  |  |  |
| ≥ 10 g/dL | 65,3 | 61,4 | 58,8 | 52,1 | 52,1 |
| < 10 g/dL | 54,1 | 52,6 | 48,8 | 45,4 | 30,1 |
| Underweight |  |  |  |  |  |
| No | 70,5 | 67 | 64,4 | 57,8 | 15,5 |
| Yes | 10,3 | 6,9 | 3,4 | 2,3 | 2,3 |
| Opportunist disease |  |  |  |  |  |
| No | 76,6 | 72,4 | 69,5 | 62,9 | 15,2 |
| Yes | 39 | 36,7 | 33,7 | 28,8 | 14,5 |
